# Divergent immunometabolic reprogramming in psoriasis and atopic dermatitis: a tale of two inflammatory skin diseases

**DOI:** 10.3389/fimmu.2026.1879634

**Published:** 2026-07-16

**Authors:** Nan Chen, Yehong Yue, Liang Zhou

**Affiliations:** 1 Department of Dermatology, Xuancheng People’s Hospital, Xuancheng, China; 2 Department of Infectious Disease, Shaoyang Central Hospital, Shaoyang, China; 3 Department of General Medicine, the Second Affiliated Hospital of Wannan Medical College, Wuhu, China

**Keywords:** AhR, atopic dermatitis, glycolysis, HIF-1α, immunometabolism, lipid metabolism, metabolic reprogramming, mTOR

## Abstract

Psoriasis (PSO) and atopic dermatitis (AD) are the two most common chronic inflammatory skin diseases in clinical practice. For a long time, they have been classified in pathological immunology under the opposing model of “Th17/IL-17 vs. Th2/IL-4-IL-13.” However, over the past decade, with the rise of immunometabolism, our understanding of chronic inflammatory diseases has undergone a significant shift. Metabolic pathways in immune cells are no longer viewed merely as auxiliary systems providing energy, but rather as core regulatory networks that determine cellular differentiation and effector functions. In psoriasis, hallmark features include upregulation of glycolysis, activation of hypoxia-inducible factor-1α (HIF-1α), abnormalities in cholesterol metabolism, and Warburg-like metabolism in keratinocytes. In AD, defects in epidermal ceramide synthesis, impaired essential fatty acid metabolism, disruption of the tryptophan-kynurenine pathway, and abnormal aromatics receptor (AhR) signaling constitute its unique metabolic profile. This article systematically reviews the distinct pathways of metabolic reprogramming in keratinocytes and immune cells in psoriasis and AD, and summarizes the mechanisms of action of key molecules such as mTOR and AMPK. Furthermore, it explores the differential therapeutic potential of metabolism-targeted drugs, including metformin, statins, PPARγ agonists, and AhR modulators. Furthermore, the expression of common regulatory factors such as HIF-1α, PPARγ, and SREBP exhibits opposite trends in the two diseases. Finally, the article discusses the differential therapeutic potential of these metabolite-targeted drugs. This paper compares psoriasis and AD within a unified metabolic immunology framework, aiming to elucidate how these two diseases evolve from similar inflammatory responses into conditions with distinctly different clinical manifestations, and to provide a new theoretical foundation for future metabolism-based personalized therapies.

## 1 Introduction

The global prevalence of psoriasis is approximately 2–3%. It is characterized by excessive epidermal proliferation, abnormal epidermal differentiation, and IL-17/IL-23 axis-mediated inflammatory infiltration in the dermis (1–3). Clinically, typical lesions present as thick-walled, silvery-white, scaly erythematous patches, often accompanied by systemic complications such as joint, cardiovascular, and metabolic syndrome-related issues (4–7). Atopic dermatitis (AD) is another chronic inflammatory skin disease with a high burden; its prevalence reaches 15–20% in children and remains between 7–10% in adults (8–10). The core manifestations of AD include disruption of the skin barrier, dry skin, intense pruritus, and recurrent eczematous lesions; immunologically, it is characterized by a Th2-dominant profile, high expression of IL-4 and IL-13, elevated IgE levels, and eosinophilic infiltration (11–14).

Although both conditions are chronic T-cell-mediated skin inflammations, their molecular pathologies diverge along several key axes, although the two diseases are not strictly opposite in every molecular feature (15–18): IL-17A, IL-22, and IFN-γ dominate in psoriasis, whereas IL-4, IL-13, and IL-31 dominate in AD; in psoriasis, keratinocytes undergo excessive proliferation, whereas in AD, keratinocyte differentiation is impaired and the skin barrier is compromised. Both diseases nonetheless share a Th22/IL-22 component and a degree of mixed-cytokine overlap, so the contrast is one of dominant polarity rather than absolute opposition. Clinically, biologics targeting IL-17 and IL-23 have completely transformed the treatment landscape for psoriasis (19–22), while dupilumab, tralokinumab, and JAK inhibitors have become the mainstream targeted therapies for AD (23–27). This contrast makes any comparative study seeking commonalities and differences between the two diseases particularly meaningful (28).

Beyond their immunological contrast, psoriasis and AD can also be separated on histopathology, and these structural differences foreshadow the metabolic divergence developed in the following sections. The two diseases share a common backbone: both are chronic, T-cell-driven dermatoses that show epidermal acanthosis, focal parakeratosis, and a perivascular lymphocytic infiltrate in the papillary dermis (1–3, 11–14). Their differences, however, are diagnostic. Psoriatic plaques display regular acanthosis with elongated rete ridges, loss of the granular layer, neutrophil collections in the cornified and spinous layers (Munro microabscesses and the spongiform pustules of Kogoj), and dilated, tortuous capillaries in the dermal papillae; despite this florid hyperproliferation the permeability barrier remains comparatively preserved (1–3, 29, 30). AD lesions are instead dominated by spongiosis in the acute phase and by lichenification in chronic plaques, with eosinophils accompanying the lymphocytic infiltrate. Its defining functional abnormality is lipidic rather than proliferative: the stratum corneum shows a marked reduction in total ceramide, a shift from long-chain (C24–C26) toward short-chain species, and a lower free-fatty-acid content, so that the lipid “mortar” of the barrier is left incomplete (31–38). From a pathological standpoint, then, AD is characterised above all by a significant reduction in epidermal lipid metabolism, whereas psoriasis is defined by keratinocyte hyperproliferation against a relatively intact lipid barrier. This histological split maps directly onto the metabolic one examined below: a glycolytic, hyperproliferative, angiogenic program in psoriasis set against a lipid-deficient, barrier-poor program in AD. Reading the two diseases through both lenses at once gives the metabolic heterogeneity discussed in this review a concrete, disease-specific anchor.

Over the past decade, immunometabolism has revealed a core fact: the metabolic pathways of T lymphocytes, myeloid cells, and even non-immune cells not only meet energy demands but also actively shape cellular phenotypes and effector functions (39–48). Resting T cells primarily rely on oxidative phosphorylation (OXPHOS) and fatty acid oxidation (FAO) to maintain homeostasis; once activated by TCR and co-stimulatory signals, cells rapidly switch to an anabolic state dominated by glycolysis and glutamine metabolism (49–53). This switch is synergistically regulated by signaling nodes such as PI3K-AKT-mTOR, AMPK, HIF-1α, and Myc (54–58).

The metabolic preferences of different T cell subsets have been systematically characterized (59–62): effector T cells and Th17 cells favor glycolysis; regulatory T cells (Tregs) and memory T cells favor FAO and OXPHOS; Th2 cells rely on more active lipid synthesis and moderate glycolysis, but their dependence on mTORC2 differs from that of Th17 cells (63–66). These findings raise a clinically relevant question: In Th17-dominant psoriasis and Th2-dominant AD, does the body’s metabolic reprogramming also exhibit corresponding divergences?

Existing reviews on metabolic immunology either focus solely on psoriasis (48, 67, 68) or discuss only lipid metabolism and the barrier in AD (69–71). Although several studies have directly compared the two diseases at the transcriptomic, proteomic, and immunologic levels (15–18, 72), an integrated synthesis that organises these comparisons around their shared metabolic regulators—and that spans keratinocytes, T-cell subsets, myeloid cells, and innate lymphoid cells in a single framework—is still lacking. It is precisely this kind of comparison that can reveal patterns not visible in single-disease studies. For example, HIF-1α is upregulated in both psoriatic keratinocytes and Th17 cells and promotes glycolysis (73–75), whereas its role in AD is more nuanced, potentially related to barrier repair and keratinocyte differentiation (76); mTOR is significantly activated in psoriatic lesions (77, 78), but in AD, the role of mTOR has been reported to be bidirectional—exerting both pro-differentiatory effects and participating in Th2 polarization (79, 80).

**Figure 1:**
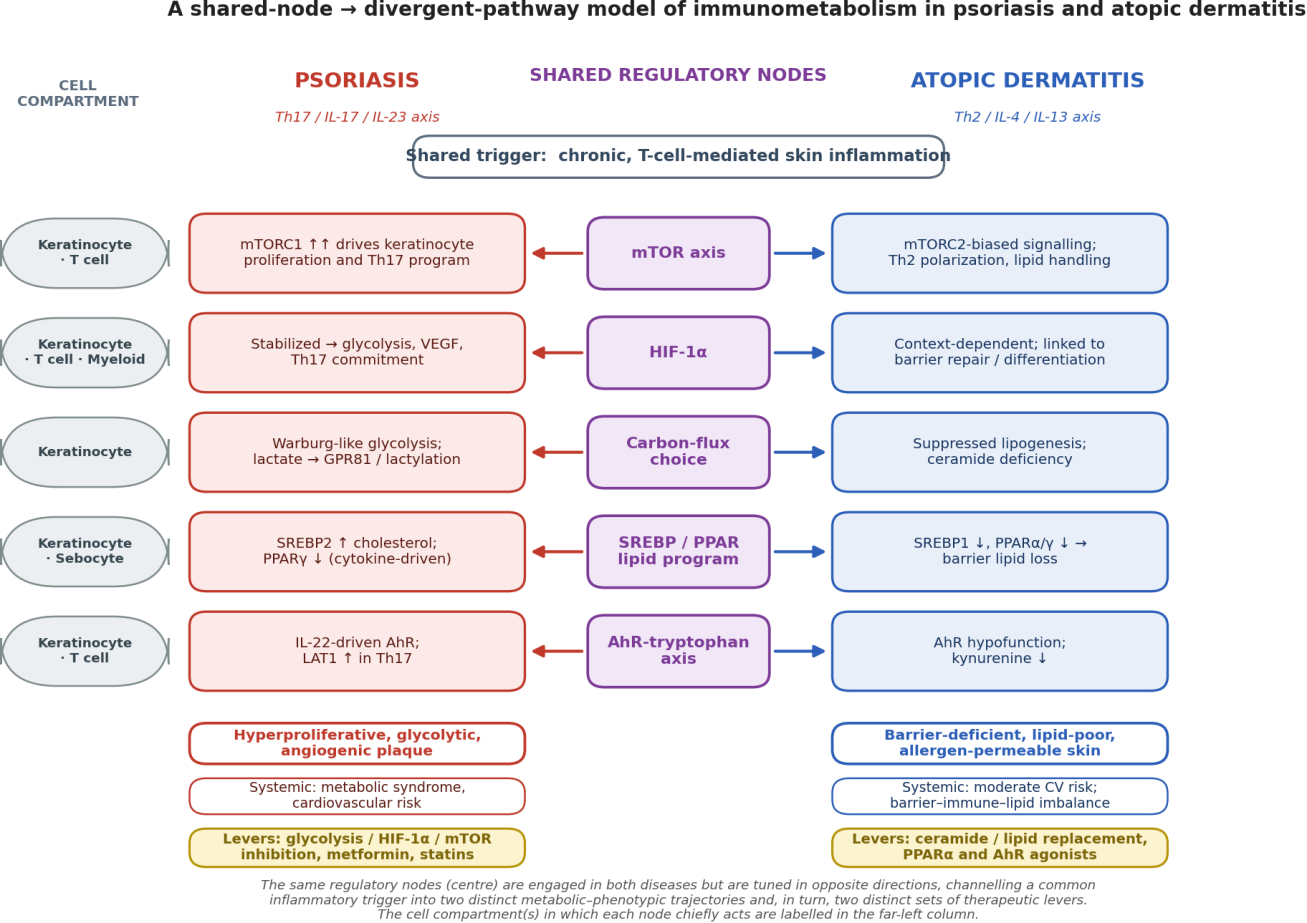
A shared-node to divergent-pathway model of immunometabolism in psoriasis and atopic dermatitis. A common trigger (chronic, T-cell-mediated skin inflammation, top) engages a central set of shared regulatory nodes—the mTOR axis, HIF-1α, the carbon-flux (glycolysis vs. lipid) decision, the SREBP/PPAR lipid program, and the AhR–tryptophan axis. Each node is tuned in opposite directions in the two diseases (red arrows, psoriasis, left; blue arrows, AD, right), channelling the same trigger into two distinct metabolic–phenotypic trajectories and, ultimately, two distinct sets of therapeutic levers (bottom). The figure is intended to convey the logical relationships among nodes rather than to enumerate individual findings.

Putting these seemingly disparate findings together allows us to outline a bifurcated model of “shared metabolic nodes—divergent pathways.” A wide range of metabolic markers has been reported in the two diseases, including the glycolytic enzymes (GLUT1, HK2, PFKFB3, PKM2, LDHA), the lactate sensor GPR81, the oxygen sensor HIF-1α, the mTORC1/mTORC2 and AMPK energy-sensing axes, the lipogenic regulators SREBP1/2, SCD1 and the ELOVL family, the ceramide-synthesis machinery, the nuclear receptors PPARα/γ, the AhR–tryptophan–kynurenine axis, and selected amino-acid transporters such as LAT1. Rather than catalogue each of these in isolation, we use the comparison to narrow the list to the nodes at which the two diseases diverge most consistently—principally HIF-1α and the glycolytic program, the mTOR axis, and the SREBP/PPAR-controlled lipid program, with AhR acting as a shared but oppositely tuned hub. Using this framework, this review systematically analyzes the metabolic characteristics of key players—including keratinocytes, T-cell subsets, myeloid cells, and ILCs—and discusses the implications of these differences for therapeutic strategies (**Figure 1**).

## 2 Metabolic divergence in keratinocytes glycolysis-dominated vs. lipid metabolism imbalance

The central message of this section is that psoriatic and AD keratinocytes are not simply more- or less-inflamed versions of the same cell; they sit at opposite ends of a single metabolic decision—whether carbon is committed to glycolysis or to lipid synthesis. Psoriatic keratinocytes divert glucose into aerobic glycolysis and lactate, a Warburg-like state that fuels rapid proliferation, whereas AD keratinocytes fail to sustain the lipogenic program required to build the stratum-corneum barrier. The two subsections below are deliberately organised around this contrast: Section 2.1 describes the glycolytic gain-of-function in psoriasis and Section 2.2 the lipogenic loss-of-function in AD, so that the divergence in energy-substrate choice can be read off directly.

### 2.1 Psoriatic keratinocytes: upregulated glycolysis and Warburg-Like metabolism

Keratinocytes in psoriatic lesions exhibit marked increases in proliferative activity, shortened epidermal transit time, and abnormal expression patterns of terminal differentiation markers (filaggrin, loricrin, and involucrin) (29, 30). Metabolomic and transcriptomic studies consistently show that the expression of key glycolytic enzymes (HK2, PFKFB3, PKM2, LDHA, GLUT1/SLC2A1) is upregulated in psoriatic lesions, leading to increased lactate production (81–84). This phenomenon bears a strong molecular resemblance to the Warburg effect observed in tumor cells—even under aerobic conditions, keratinocytes preferentially metabolize glucose into lactate (85, 86).

Mechanistically, IL-17A and IL-22 induce the expression of glycolytic enzymes such as HK2 and PFKFB3 via the STAT3 and NF-κB pathways and stabilize the HIF-1α protein (73, 87, 88). HIF-1α, in turn, enhances glycolysis, stimulates VEGF release, and promotes angiogenesis, which is consistent with the histological finding of excessive vascularization in psoriasis (89–92). Targeting HK2, PKM2, or PFKFB3 significantly alleviates epidermal hyperplasia and inflammation in a mouse imiquimod (IMQ)-induced psoriasiform model (84, 93–95).

It is worth noting that lactate is not only a metabolic end product but also an important signaling molecule. Histone lactylation, as a newly discovered epigenetic modification (96), has been shown to regulate macrophage polarization and immune homeostasis. Recent studies suggest that lactate can promote the IL-23/IL-17 pathway via the GPR81 receptor, exacerbating psoriasiform inflammation (97, 98).

### 2.2 AD keratinocytes: lipid metabolic imbalance and barrier defects

The most prominent metabolic abnormality in AD keratinocytes is the disruption of lipid metabolism (31, 99, 100). As early as 1991, Imokawa et al. reported reduced ceramide levels in the stratum corneum of AD patients (31); Subsequent studies have confirmed a decrease in total stratum corneum ceramide levels and the proportion of long-chain ceramides (C24–C26), along with an increase in short-chain ceramides (32–34, 71). This lipidomic profile results in an incomplete “brick-and-mortar” structure of the stratum corneum, increased water loss, and easier penetration by exogenous allergens and microorganisms (35–38).

Mechanistically, IL-4 and IL-13 downregulate the expression of key enzymes in lipid synthesis—members of the SCD (stearoyl-CoA desaturase) and ELOVL (elongation of very long-chain fatty acids) families—via the STAT6 pathway (33, 69, 101). Berdyshev et al. directly confirmed via lipidomics that long-chain ceramides are reduced and short-chain ceramides are increased in AD lesions, attributing this change to Th2 cytokines (33). Furthermore, loss-of-function mutations in the filaggrin (FLG) gene are one of the most important genetic susceptibility factors for AD (102, 103); the reduction of filaggrin degradation products (trans-urocanic acid, pyrrolidone carboxylic acid) further exacerbates pH disruption, barrier damage, and inflammation (104, 105).

More importantly, energy metabolism in AD keratinocytes is also abnormal but in the opposite direction: unlike in psoriasis, the expression of genes related to glycolysis is not significantly elevated in AD lesions; instead, fatty acid synthesis-related pathways (SREBP, SCD1, FASN) and PPARα/γ signaling are suppressed (69, 101, 106). This implies that AD keratinocytes are in a state of “lipid deficiency,” making it difficult to maintain the lipid synthesis required for the stratum corneum barrier.

### 2.3 Comparisons and critical nodes

**Figure 2:**
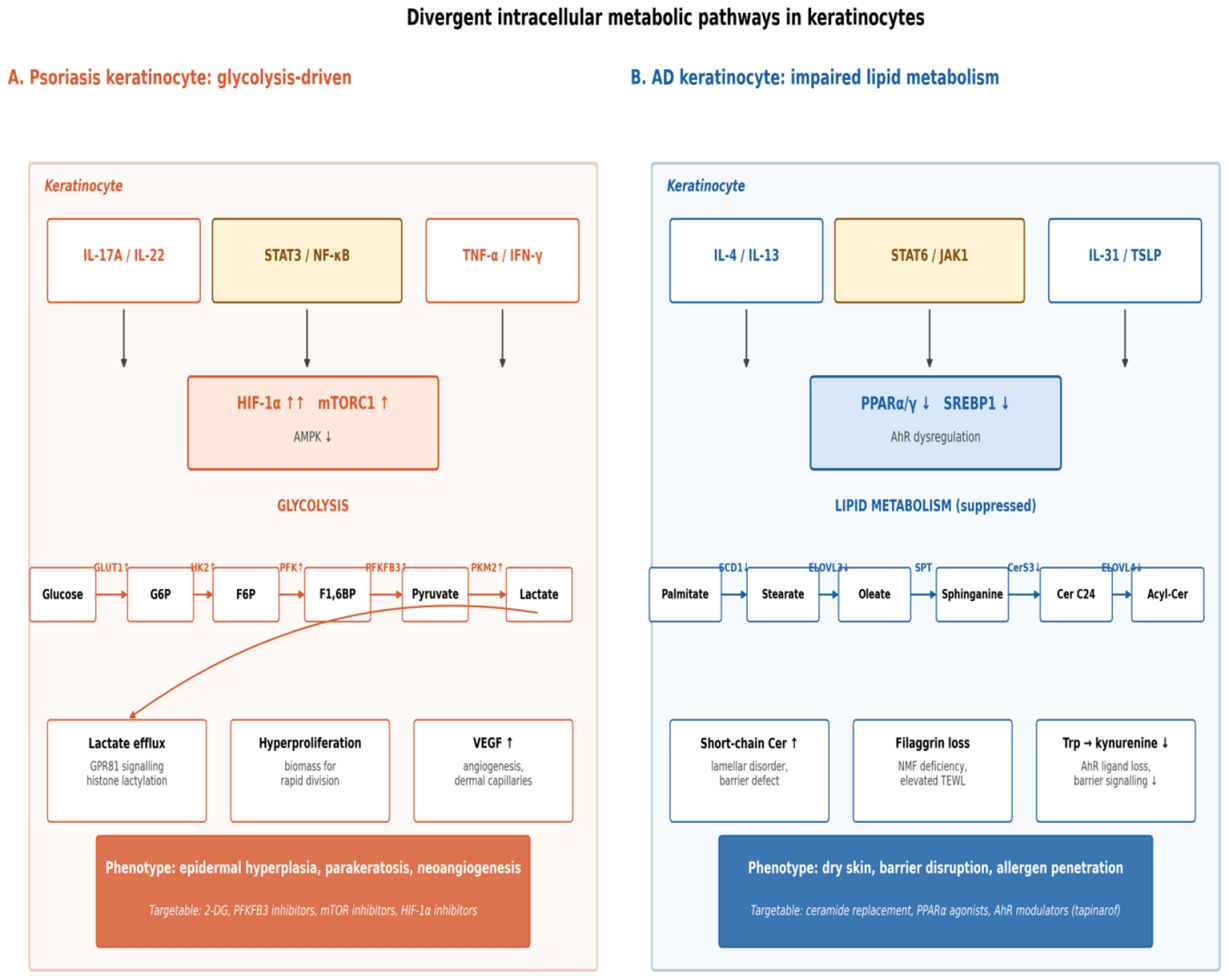
Divergent intracellular metabolic pathways in keratinocytes. **(A)** In psoriasis, IL-17A/IL-22 and TNF-α drive STAT3/NF-κB activation, HIF-1α stabilisation and mTORC1 hyperactivation, which together push the keratinocyte into a glycolysis-dominated, Warburg-like state. **(B)** In AD, IL-4/IL-13 signalling through STAT6/JAK1 suppresses SREBP1 and PPARα/γ, blunting the SCD1/ELOVL-driven ceramide synthesis pathway that underlies barrier dysfunction.

A side-by-side comparison of the metabolic profiles of keratinocytes from the two diseases reveals that psoriasis keratinocytes are characterized by “high glycolysis, high lactate, high HIF-1α, active proliferation, but a relatively intact barrier”; in contrast, atopic dermatitis (AD) keratinocytes exhibit “low lipid synthesis, ceramide deficiency, PPARα/γ inhibition, and barrier disruption.” HIF-1α, AhR, and SREBP are several key nodes where the metabolic pathways diverge significantly between the two conditions (**Figure 2**). This metabolic divergence is closely associated with the distinct cytokine environments of the two diseases (IL-17/IL-22 vs. IL-4/IL-13) and may be a direct result of the corresponding immune microenvironment driving the metabolic remodeling of keratinocytes.

### 2.4 Sebaceous glands: a second, disease-divergent source of skin lipids

Keratinocytes are not the only epithelial cells that shape the cutaneous lipid environment. Sebaceous glands supply a large fraction of the skin-surface lipid film, and their alteration is increasingly recognised across inflammatory dermatoses (107). Recent spatial-transcriptomic profiling of lesional and non-lesional human skin showed that sebaceous glands in both psoriasis and AD express the core lipid-handling machinery (FASN, the SCD/FADS family, PPARG, FABP7, APOC1) but diverge in a disease-specific way: in AD the differentially expressed genes cluster around lipid metabolism (for example ACAD8, FADS6, EBP) together with the Th2-linked, lipid-regulating enzyme HSD3B1, whereas in psoriasis the sebaceous compartment is dominated by inflammation- and interferon-related transcripts (for example SERPINF1, IFIT1/3, DDX58) (108, 109). This pattern mirrors the keratinocyte divergence described above—an AD-side emphasis on lipid and barrier biology versus a psoriasis-side emphasis on inflammatory and glycolytic programs—and shows that the lipid deficit in AD is not confined to keratinocyte ceramide synthesis but extends to glandular sebum production. Sebaceous involvement therefore adds a second, independent axis to the lipid-versus-glycolysis contrast and is a reminder that the metabolic divergence operates at the level of the whole pilosebaceous unit, not the keratinocyte alone. Detailed metabolic-flux data for sebocytes in the two diseases are still sparse, which we regard as a concrete gap for future work.

## 3 Metabolic preferences of T cell subpopulations: the Th17 vs. Th2 divergence

It is most informative to compare Th17 (psoriasis-relevant) and Th2 (AD-relevant) cells one pathway at a time rather than one cell type at a time. Two pathways carry most of the divergence. First, glycolysis: Th17 differentiation is strongly glycolytic and HIF-1α-dependent—2-DG-sensitive and sustained by mTORC1—whereas Th2 cells are only moderately glycolytic, rely more on oxidative phosphorylation, and are largely HIF-1α-independent (HIF-1α can even oppose Th2 commitment). Second, lipid metabolism: although both subsets use the lipogenic enzyme ACC1, they use it differently—in Th17 cells *de novo* fatty-acid and SREBP2-driven cholesterol synthesis supports proliferation and RORγt activity, whereas in Th2 cells PPARγ-dependent lipid handling and fatty-acid oxidation support IL-4-driven effector function. Read in parallel, the picture is a glycolysis-versus-lipid split that closely parallels the keratinocyte divergence of Section 2. The subsections that follow present the evidence for each pathway in turn, beginning with glycolysis and the HIF-1α/mTOR axis (Section 3.1) and then lipid metabolism (Sections 3.1–3.2), before turning to Tregs (Section 3.3) and amino-acid metabolism (Section 3.4).

### 3.1 Metabolic characteristics of Th17 cells: glycolysis-HIF-1α dependence

The metabolic preferences of Th17 cells have been relatively well characterized (65, 110–112). The seminal work by Shi et al. demonstrated that HIF-1α is a key metabolic transcription factor for Th17 differentiation; its inactivation significantly inhibits Th17 differentiation while promoting Treg differentiation (113). Dang et al. further revealed that HIF-1α acts as a switch in the Th17/Treg balance by promoting RORγt expression and FoxP3 degradation (114). Metabolically, Th17 cells are highly dependent on glycolysis and are extremely sensitive to 2-DG (2-deoxy-D-glucose); mTORC1 maintains their glycolytic state via 4E-BP1/eIF4E and HIF-1α (55, 115). Consistent with these mechanistic studies, direct metabolic analysis of T cells in psoriatic lesions—including scRNA-seq and flow-cytometric metabolic profiling—has confirmed high glycolytic activity, high HIF-1α expression, and high mTOR activity in lesional Th17 cells (116–118), and strategies targeting glycolysis (2-DG, PFKFB3 inhibitors), HIF-1α, and mTOR (rapamycin) are all effective in the imiquimod (IMQ) model (84, 93, 119). (Text relocated here from the former third paragraph of this section to keep the glycolysis–HIF-1α–mTOR evidence together).

Lipid metabolism is also involved in Th17 fate determination. Berod et al. found that *de novo* fatty acid synthesis mediated by acetyl-CoA carboxylase 1 (ACC1) is essential for Th17 differentiation; inhibition or knockout of ACC1 specifically suppresses Th17 differentiation and redirects it toward Tregs (120). Cholesterol metabolic pathways are also involved: Hu et al. demonstrated that SREBP2-mediated cholesterol synthesis activates RORγt function, while Santori et al. reported that the cholesterol derivative desmosterol acts as an endogenous ligand for RORγt (121, 122).

### 3.2 Metabolic characteristics of Th2 cells: a flexible combination of oxidative phosphorylation and lipid metabolism

Compared to Th17 cells, the metabolic preferences of Th2 cells have long been overlooked; recent studies have begun to fill this gap (63, 64). Nobs et al. found that Th2 cells rely on PPARγ-mediated lipid metabolism to maintain their effector functions: deletion of PPARγ significantly attenuated Th2-mediated allergic airway inflammation (123). Similarly, Endo et al. demonstrated that the lipid metabolic enzyme ACC1 plays a crucial role in Th2 differentiation, though its mechanism of action differs from that in Th17 cells—where ACC1 promotes membrane lipid synthesis to support rapid proliferation—while in Th2 cells, ACC1 is associated with IL-4 production (120, 124).

Th2 cells are more dependent on mTORC2 than on mTORC1, and their differentiation is impaired when the PI3K-Akt-mTORC2 pathway is absent (125, 126). The role of HIF-1α in Th2 differentiation is relatively limited; some studies even show that HIF-1α inhibits Th2 differentiation (76), which stands in stark contrast to the high dependence of Th17 cells on HIF-1α.

**Figure 3:**
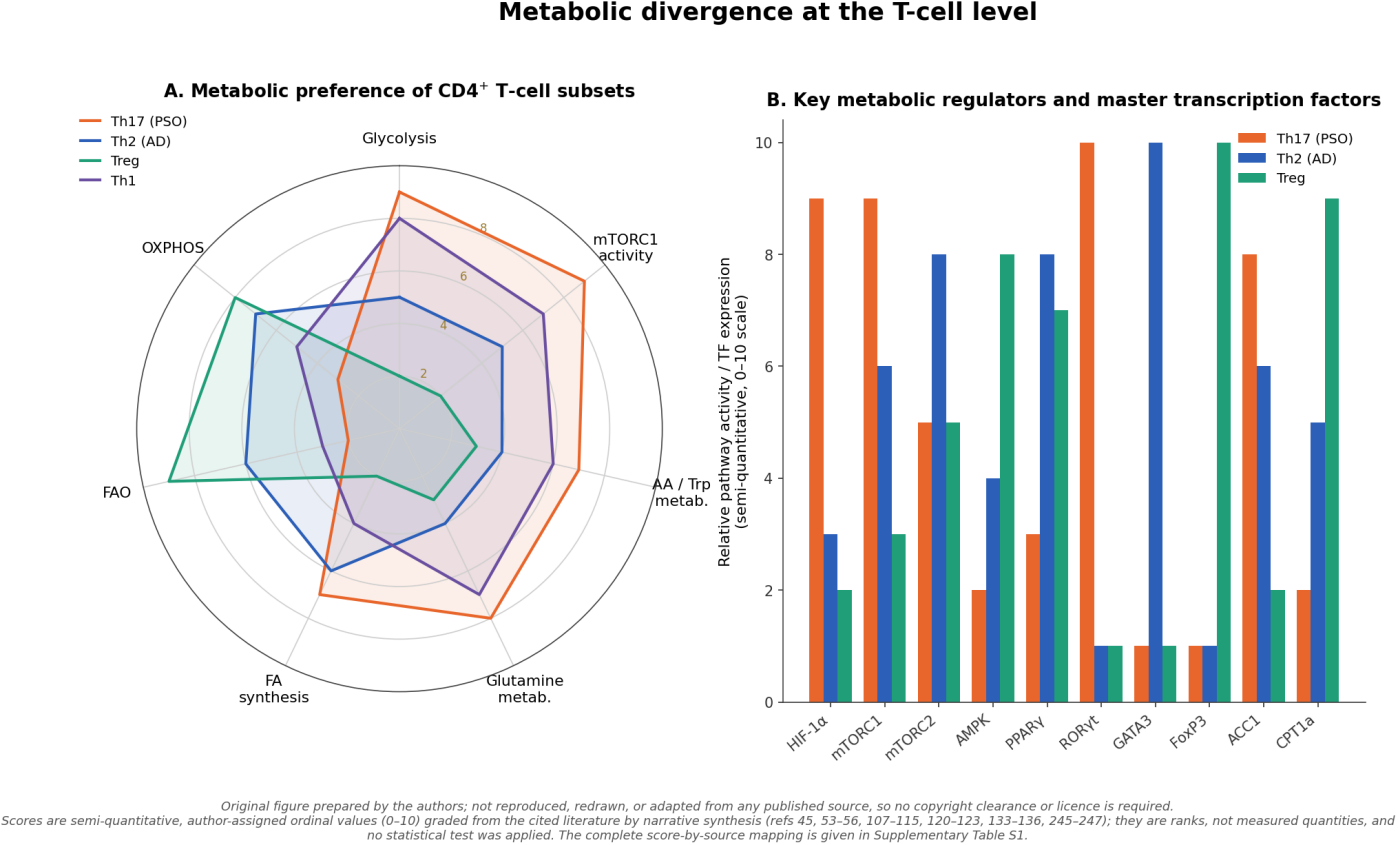
Metabolic divergence at the T-cell level. **(A)** Radar chart of the metabolic preferences of CD4+ T-cell subsets across glycolysis, OXPHOS, FAO, fatty-acid synthesis, glutamine and amino-acid metabolism, and mTORC1 activity. **(B)** Relative expression of key metabolic regulators and master transcription factors across Th17 (PSO-relevant), Th2 (AD-relevant) and Treg subsets. The values in both panels are semi-quantitative, author-assigned scores (0–10) that summarise the relative, qualitative tendencies reported in the literature rather than measured quantities; they were synthesised from refs 45, 53–56, 107–115, 120–123, 133–136 and 245–247 and are intended only for comparison across subsets. The corresponding source statement is reproduced as a note within the figure itself. This figure is an original schematic prepared by the authors for this review; it is not reproduced, redrawn, or adapted from any published figure, so no third-party copyright clearance or licence is required. No primary numerical dataset underlies the panels. The scores were assigned by narrative synthesis: for each subset (Th17, Th2, Treg, Th1) and each metabolic axis we read the cited primary and review sources, graded the reported tendency on a five-anchor ordinal scale (0 = absent/strongly suppressed, 2–3 = low, 5 = intermediate, 7–8 = high, 10 = dominant/defining), and resolved the few discordant cases by consensus among the authors. The grades are therefore ranks, not measurements, and no statistical test was applied to them. The full score-by-source mapping is provided in **Supplementary Table 1** so that each value can be traced back to the reference(s) that justify it.

In AD lesions, infiltrating Th2 and Th22 cells exhibit active oxidative phosphorylation and high expression of lipid synthesis genes (127, 128). It is worth noting that in addition to Th2 cells, Th22 and Th17 cells (particularly in Asian populations and pediatric AD) are also involved in AD (129–132), suggesting that the metabolic profile of AD may be more complex than the traditionally recognized “single Th2” model. The differences between the two conditions at the T-cell metabolic level are shown in **Figure 3**. The panels in **Figure 3** are an original synthesis rather than reproduced data; the values are author-assigned ordinal scores graded from the cited literature, and the complete score-by-source mapping is provided in **Supplementary Table 1**.

### 3.3 Tregs and metabolic homeostasis: a common metabolic dysregulation in both diseases

Abnormalities in Treg numbers or function have been reported in both diseases (133–135). Metabolically, Tregs prefer fatty acid oxidation (FAO) and oxidative phosphorylation (OXPHOS), with their mitochondrial function maintained by FoxP3 and PGC-1α (136, 137). AMPK activation promotes Treg differentiation, whereas excessive mTORC1 activation suppresses Tregs (138, 139). In psoriatic lesions, where a high-mTOR, high-glycolysis microenvironment is well documented, this tone is expected to destabilise local Tregs. Whether an equivalent metabolic state operates in AD lesions is not yet established: direct measurements of lesional mTOR activity and glycolytic flux in AD are still lacking. We therefore restrict the firm claim to psoriasis and treat any shared “metabolic–immune regulatory imbalance” in AD as a hypothesis to be tested rather than an established feature.

### 3.4 Amino-acid metabolism: a shared dependency with subset-specific tuning

Beyond glucose and lipids, amino-acid handling is a third metabolic layer that separates effector from regulatory T cells and is relevant to both diseases. Antigen-receptor engagement upregulates the System L transporter SLC7A5 (LAT1); loss of SLC7A5 abolishes the mTORC1- and Myc-dependent metabolic switch required for Th1 and Th17 differentiation while sparing Treg induction (61). Glutaminase-dependent glutamine catabolism is similarly bifunctional—it is required for Th17 differentiation but constrains Th1/CTL effector programs, so its blockade affects these subsets in opposite directions (140)—and arginine availability reprograms T cells toward a memory-like, oxidative state that favours survival (141, 142). These observations map onto the disease comparison. The Th17-skewed, mTOR-high environment of psoriatic lesions is expected to depend heavily on LAT1-mediated large-neutral-amino-acid uptake and on glutaminolysis; consistent with this, CD69-controlled LAT1 uptake of tryptophan and AhR-dependent IL-22 secretion have been demonstrated directly in psoriatic skin (143) (see Section 5). The Th2 program of AD appears comparatively less glutamine- and LAT1-dependent, in keeping with its lower glycolytic and mTORC1 tone. Amino-acid transporters and glutaminase are therefore plausible—and so far under-explored—points of metabolic divergence and of potential intervention; head-to-head measurements in psoriatic versus AD lesions are not yet available.

## 4 Metabolic reprogramming of myeloid cells, DCs, and ILCs

The core point of this section is that the innate and myeloid compartments follow the same glycolysis-versus-lipid divergence already established for keratinocytes and T cells: psoriasis is dominated by glycolytic, M1-like macrophages, glycolytic DCs/pDCs, and ILC3, whereas AD is dominated by lipid-/OXPHOS-oriented M2-like macrophages, IDECs, and lipid-droplet-dependent ILC2. The subsections below give the evidence for each cell type in turn.

### 4.1 Macrophage polarization and metabolism

Macrophage polarization is closely linked to metabolism: M1 macrophages prefer glycolysis, the pentose phosphate pathway, and a disrupted tricarboxylic acid cycle (accompanied by accumulation of succinate and itaconic acid), while M2 cells prefer OXPHOS and FAO (144–147). Tannahill et al. revealed a HIF-1α-dependent mechanism by which succinate drives IL-1β expression (148); Mills et al. further confirmed that succinate dehydrogenase (SDH) supports the metabolic shift in pro-inflammatory macrophages (149); Lampropoulou et al. identified itaconic acid as an SDH inhibitor and a key anti-inflammatory metabolite (150). In psoriatic lesions, M1-like macrophages predominate, glycolysis is upregulated, and HIF-1α protein accumulates (151–153); in AD lesions, M2-like macrophages participate in the amplification of Th2 responses, but their metabolic characteristics remain incompletely understood (154, 155).

### 4.2 Dendritic cells and plasmacytoid DCs

The dendritic-cell compartment follows the same glycolysis-versus-lipid logic seen in keratinocytes and T cells. On TLR-driven maturation, conventional DCs switch rapidly to glycolysis and increase lipid synthesis to fuel membrane expansion and cytokine output (156, 157). In psoriasis this glycolytic program dominates: lesional conventional DCs (cDC2) and plasmacytoid DCs (pDCs) are glycolysis-dependent (158, 159), and pDCs—which help initiate disease through type-I interferon (IFN-α) (160, 161)—meet that demand through mTOR-driven glycolysis (162). In AD the principal antigen-presenting population differs: inflammatory dendritic epidermal cells (IDECs) present allergen through the FcϵRI–IgE axis, and their metabolism is skewed toward fatty-acid oxidation and oxidative phosphorylation rather than glycolysis (163, 164). At the level of innate antigen-presenting cells, then, the DC compartment reproduces the glycolytic (psoriasis) versus oxidative/lipid (AD) split established above.

### 4.3 ILC2 and lipid metabolism

Type 2 innate lymphoid cells (ILC2) are key participants in AD (165, 166). Wilhelm et al. demonstrated that ILC2 function depends on lipid droplet formation and fatty acid oxidation, and that exogenous fatty acids can amplify their IL-13 production (167); Karagiannis et al. further revealed that lipid droplet metabolism regulates the pathogenicity of ILC2 in airway inflammation (168). These findings suggest a possible bidirectional feedback loop between ILC2 and lipid metabolism abnormalities in AD. Although ILC3 (IL-17/IL-22-producing) cells are present in psoriasis, their metabolic characteristics remain less well defined than those of ILC2 (169).

## 5 Metabolic interactions between keratinocytes and immune cells

The clearest example of metabolite-mediated cross-talk that distinguishes the two diseases is the tryptophan–kynurenine pathway and its downstream activation of the aryl-hydrocarbon receptor (AhR) (143, 170, 171). IDO1 and TDO mediate the degradation of tryptophan to kynurenine, which acts as an endogenous ligand for AhR to regulate immune balance. Abnormal AhR signaling in AD lesions has been widely reported (172–174); some studies have found abnormal IDO1 expression and a decreased kynurenine/tryptophan ratio in AD lesions (175, 176). The AhR agonist tapinarof has demonstrated efficacy in both moderate-to-severe AD and psoriasis, suggesting that AhR is a key node connecting the metabolic-immune axis of these two diseases (177–180).

## 6 Branching pathways of shared metabolic nodes

The central argument of this review is that a small set of regulatory nodes is shared by both diseases but is tuned in opposite directions, and these nodes are summarised side by side in **Table 1**. Five of them deserve individual discussion: mTOR, AMPK, HIF-1α, the PPARγ/PPARα receptors, and SREBP. For each, we first state the direction of the divergence and then give the supporting evidence.

**Table 1:** Comparison of metabolic features between psoriasis and atopic dermatitis (AD).

Feature	Psoriasis (PSO)	Atopic dermatitis (AD)
Dominant Th subset	Th17/Th22/Th1	Th2/Th22 (with possible Th17)
Keratinocyte glycolysis	↑↑ significantly upregulated (HK2, PFKFB3, PKM2)	No significant upregulation
Keratinocyte lipid synthesis	Relatively preserved	↓↓ significantly inhibited (SCD1, ELOVL, ceramide)
HIF-1α	Pro-inflammatory driver	Variable/barrier-related
mTORC1	↑↑ significantly activated	↑ (biased toward mTORC2)
AMPK	Variable/partially downregulated	—
PPARγ/PPARα	Both downregulated (KC)	Both downregulated (KC)
SREBP1/SREBP2	SREBP2↑ cholesterol metabolism disorder	SREBP1↓ (cytokine-driven)
AhR pathway	Activated by IL-17/IL-22	Hypofunctional/kynurenine↓
Lactate/GPR81	↑↑ in lesions	Not significant
Tryptophan–kynurenine pathway	Variable: LAT1↑ in Th17	Imbalanced: IDO1↓
Dominant ILC subset	ILC3	ILC2 (lipid droplet dependent)
Macrophage polarization	M1-skewed, glycolytic type	M2-skewed
Systemic comorbidities	Metabolic syndrome, cardiovascular disease	Moderate cardiovascular risk; barrier-related
Targetable hubs	Glycolysis, HIF-1α, mTOR	Lipid metabolism, AhR, PPARα

↑ increased / up-regulated; ↑↑ markedly (significantly) increased / up-regulated; ↓ decreased / down-regulated; ↓↓ markedly (significantly) decreased / down-regulated (inhibited); — no consistent change reported or data not available / not applicable.Where an arrow is attached to a specific molecule in the cell (e.g. “SREBP2↑”, “SREBP1↓”, “kynurenine↓”, “LAT1↑”, “IDO1↓”), it indicates the change in that particular molecule; the same key applies.

### 6.1 mTOR

mTOR is significantly activated in the skin lesions of both diseases (77–80). In psoriasis, mTORC1 drives keratinocyte proliferation and Th17 polarization via 4E-BP1 and S6K1; in AD, mTOR regulates lipid metabolism and influences keratinocyte differentiation via STAT6/Th2. Rapamycin demonstrates some efficacy in models of both diseases, but the mechanisms of action are not entirely identical (181–183).

### 6.2 AMPK

AMPK acts as an energy sensor to inhibit glycolysis and promote fatty acid oxidation (FAO) and autophagy. AMPK activators (metformin, AICAR) have demonstrated anti-inflammatory and antiproliferative effects in psoriasis models (184–186); in AD models, AMPK has also been reported to improve the skin barrier (187). However, the baseline states of the AMPK pathway may differ between the two conditions: AMPK activity is significantly reduced in psoriatic lesions, suggesting a state of metabolic stress; reports on AMPK status in AD lesions are inconsistent (188).

### 6.3 HIF-1α

As previously mentioned, HIF-1α is universally elevated in psoriatic keratinocytes, Th17 cells, and myeloid cells (73, 74, 114, 153); the role of HIF-1α in AD remains controversial, with some studies suggesting that HIF-1α plays a protective role in barrier repair and keratinocyte differentiation (76), potentially in contrast to psoriasis.

### 6.4 PPARγ and PPARα

PPARγ and PPARα are key nuclear receptors involved in lipid metabolism and anti-inflammation (189, 190). PPARγ expression is downregulated in psoriatic lesions, and the agonist (pioglitazone) has a beneficial effect (191, 192); PPARα/γ expression is also reduced in AD, and PPARα agonists can promote barrier repair (193, 194). The two conditions appear to share a common direction in the PPAR pathway—both benefit from “activation”—but the specific downstream immunological mechanisms differ.

### 6.5 SREBP

SREBP1 and SREBP2 regulate fatty acid and cholesterol synthesis, respectively (195, 196). Abnormal SREBP1/2 expression and cholesterol metabolism imbalance in psoriatic lesions are associated with systemic comorbidities (atherosclerosis, metabolic syndrome) (197–199); In AD, SREBP-mediated downregulation of lipid synthesis is a key mechanism underlying barrier dysfunction (69, 101). SREBP dysregulation exhibits opposite trends in the two diseases: upregulation and dysregulation in psoriasis, and suppression in AD.

## 7 The potential for differentiated metabolic targeted therapy

**Figure 4:**
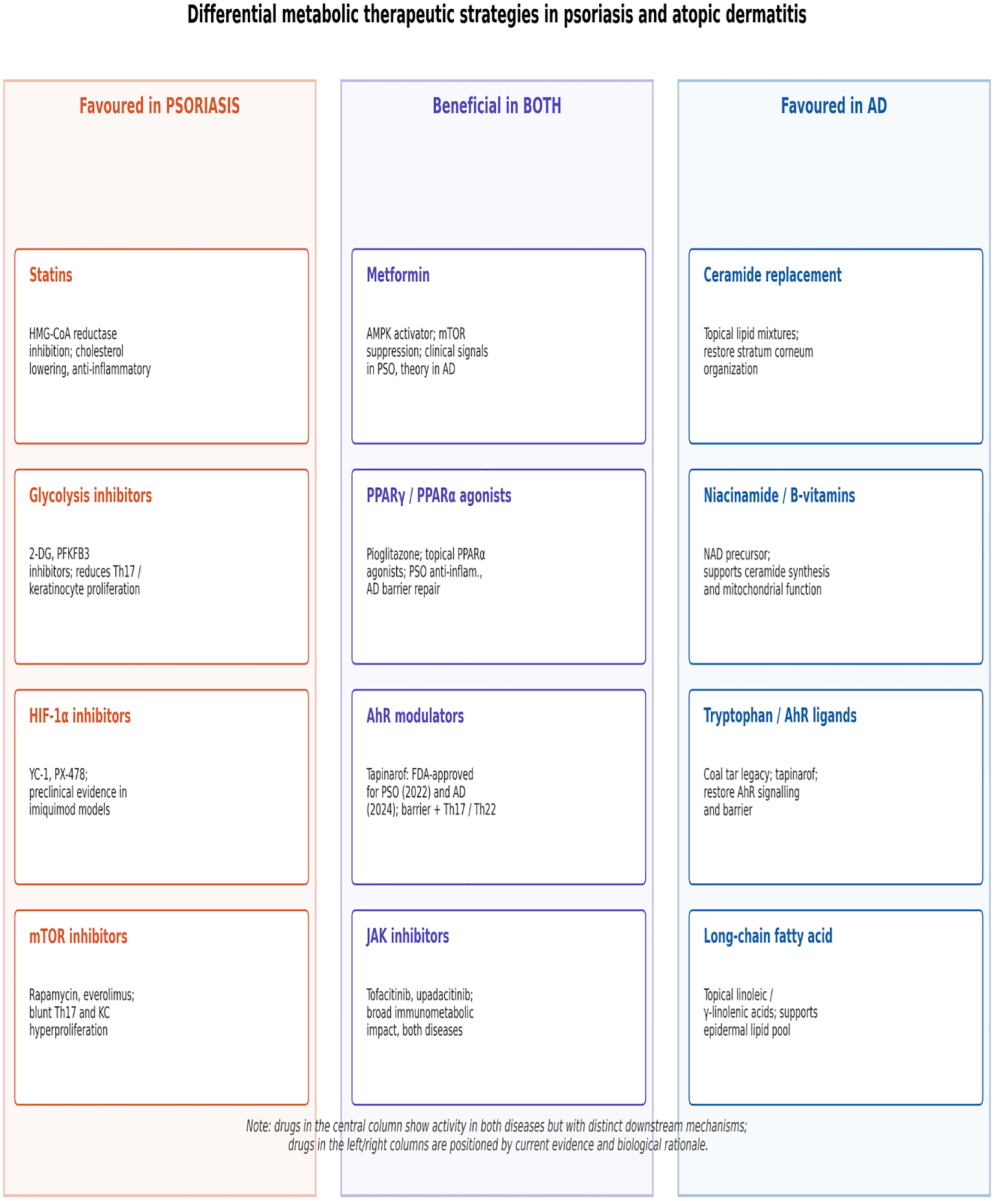
Differential metabolic therapeutic strategies. The left column lists agents favoured in psoriasis, the right column lists agents favoured in atopic dermatitis, and the central column highlights compounds with documented activity in both diseases.

The therapeutic implication of the shared-node/divergent-pathway model is direct: agents that suppress glycolysis, HIF-1α, or mTOR should favour psoriasis, agents that restore lipid synthesis and barrier function should favour AD, and a few interventions acting on nodes shared by both diseases (metformin, PPAR and AhR agonists, JAK inhibitors) can benefit either, though through different downstream mechanisms. The following subsections, together with **Figure 4** and **Table 2**, organise the candidate drugs along exactly this axis.

**Table 2:** Summary of metabolism-related clinical trials.

Drug	Mechanism	Indication	Evidence level	Reference
Metformin	AMPK activation/mTOR inhibition	Psoriasis	RCT adjunctive therapy, registry data	see main text §7.1
Pioglitazone	PPARγ agonist	Psoriasis (including PsA)	RCT placebo-controlled	see main text §7.3
Simvastatin/Atorvastatin	HMG-CoA reductase inhibition	Psoriasis	RCT preliminary trial	see main text §7.2
Sirolimus	mTORC1 inhibition	Psoriasis	RCT combination	see main text §6.1, §7
Everolimus	mTOR inhibition	Psoriasis	Case report	see main text §6.1
Tapinarof	AhR agonist	Psoriasis (FDA 2022)	Phase III (PSOARING)	see main text §7.4
Tapinarof	AhR agonist	Atopic dermatitis (FDA 2024)	Phase III (ADORING)	see main text §7.4
WBI-1001 (early tapinarof)	AhR-related	Psoriasis/AD	Phase II	see main text §7.4
Topical PPARα agonist	PPARα agonist	AD barrier repair	Preclinical preliminary trial	see main text §7.3
Topical ceramide formulation	Lipid replacement	Atopic dermatitis	Multiple RCTs	see main text §7.6
Niacinamide	NAD precursor, barrier support	Atopic dermatitis	Small trial	see main text §7.6
Dupilumab	IL-4Rα blockade (downstream metabolism)	Atopic dermatitis	Phase III	see main text §1.1
Upadacitinib/Abrocitinib	JAK inhibition	Atopic dermatitis	Phase III	see main text §1.1
Bimekizumab	IL-17A/F blockade	Psoriasis	Phase III	see main text §9
Nemolizumab	IL-31RA blockade	Atopic dermatitis	Phase IIIb	see main text §9

### 7.1 Metformin

Metformin exerts anti-inflammatory and antiproliferative effects by activating AMPK, inhibiting mTOR, and modulating glycolysis (184, 185). Epidemiological studies show that patients with psoriasis and diabetes experience improvement in skin lesions after using metformin (200, 201); multiple clinical trials have confirmed the efficacy of metformin as an adjunctive treatment for psoriasis (188, 202, 203). Although evidence is relatively limited in atopic dermatitis (AD), theoretically, AMPK activation is beneficial for barrier repair (187).

### 7.2 Statins

Statins inhibit HMG-CoA reductase and downregulate cholesterol synthesis. Psoriasis is associated with a high risk of cardiovascular disease (5, 7), and statins have been reported multiple times to improve psoriatic lesions (204–207). However, data on the effects of statins on atopic dermatitis (AD) are limited, and they may adversely affect the AD barrier by inhibiting lipid synthesis; therefore, caution is warranted (208). This is a classic example of how the “metabolic fork” guides differentiated drug therapy.

### 7.3 PPARγ and PPARα agonists

Pioglitazone has demonstrated some efficacy in small clinical trials for psoriasis (191, 192); topical PPARα agonists improve the skin barrier in AD (193, 209). Both conditions benefit from PPAR activation, but the target subtypes and dosage forms differ.

### 7.4 AhR modulators

Tapinarof, an AhR agonist, has been approved for adult psoriasis (FDA 2022) and moderate-to-severe AD (FDA 2024) (179, 180, 210). Its metabolic-immune mechanisms involve the Nrf2 antioxidant pathway, AhR-IL-17/Th17 regulation, and barrier repair (178, 211, 212).

### 7.5 Glycolysis inhibitors

2-DG, PFKFB3 inhibitors, and PKM2 modulators have shown efficacy in animal models of psoriasis (84, 93–95), but clinical translation remains in the early stages. Glycolysis inhibition is not currently considered a mainstream strategy for AD.

### 7.6 Ceramide replacement and lipid metabolism modulation

Topical synthetic ceramides, phosphatidylcholine precursors, and moisturizers containing niacinamide improve the AD barrier (213, 214); these adjunctive therapies have no clear indications in psoriasis. The potential for differentiation of metabolism-related drugs between the two conditions is summarized in **Figure 4** and **Table 2**.

## 8 The metabolic axis from a comorbidity perspective: from the skin to the system

The association between psoriasis and comorbidities such as metabolic syndrome, obesity, diabetes, and cardiovascular disease has been established (5, 7, 215–218). This comorbidity stems not only from chronic inflammation but is also associated with systemic dysregulation of the immunometabolic axis: there are multiple cross-links between IL-17 and insulin resistance, HIF-1α and atherosclerosis, and SREBP and lipid metabolism abnormalities (219–222). Systemic metabolic comorbidity in AD was once considered rare, but recent studies have begun to reveal a moderate association between AD and cardiovascular events and metabolic syndrome; in some populations, AD is also associated with obesity (223–226). The systemic metabolic abnormalities of the two diseases exhibit different patterns: psoriasis tends toward a “metabolic syndrome-like” profile, while AD leans toward “barrier-immune-lipid metabolism imbalance,” but both suggest that treatment should focus on the systemic level.

A further systemic layer that the two diseases handle differently is the gut–skin axis. Microbial metabolites, in particular the short-chain fatty acids (SCFAs) acetate, propionate, and butyrate, reach the skin from the intestinal microbiome and act through the receptors GPR43 (FFAR2) and GPR109A and through inhibition of histone deacetylases to favour Treg induction and to dampen IL-17-driven inflammation (227, 228). In psoriatic skin the SCFA-sensing receptors GPR43 and GPR109A are downregulated, and their expression can be restored by topical butyrate, linking a gut-derived metabolic signal directly to the IL-17 axis that defines psoriasis (229, 230). The gut microbiome is also dysbiotic in both diseases, but in different directions—psoriasis is associated with reduced SCFA-producing taxa and a pro-inflammatory, Th17-permissive profile, whereas AD dysbiosis (together with cutaneous Staphylococcus aureus overgrowth) is more closely tied to barrier failure and Th2 skewing. Whereas amino-acid metabolism (Section 3.4) and the tryptophan–AhR axis (Section 5) act largely within the lesion, the SCFA/gut-microbiome axis is a systemic, diet- and microbiome-modifiable input that converges on the same shared nodes, and it represents a tractable, under-used target—especially in psoriasis, where the SCFA–GPR43/109A–Treg link is best documented.

## 9 Future prospects

Metabolic immunology research on psoriasis and AD has made significant progress over the past decade, but numerous unresolved questions remain.

First, spatiotemporal heterogeneity: scRNA-seq and spatial transcriptomics have revealed extreme heterogeneity in cell types and states within skin lesions (117, 231–233). We stress that single-cell and spatial methods have already mapped many disease-specific cellular states in both psoriasis and AD, including at the sebaceous gland (109, 118, 227, 229, 231); the open gap is therefore not whether such methods can find new cell states, but that these atlases remain almost entirely transcriptional and rarely report metabolic activity. The concrete priority is to overlay metabolic readouts onto the existing maps—by inferring pathway activity from transcriptomes (for example with scMetabolism or Compass), by spatial metabolomics and mass-spectrometry imaging, and by flux measurements on sorted lesional subsets—so as to build a genuinely metabolic, rather than merely transcriptional, single-cell atlas of the two diseases.

Second, disease subtypes and personalization: AD has been subdivided into multiple phenotypes (intrinsic vs. extrinsic, Asian vs. Western, pediatric vs. adult) (132, 234–236); psoriasis also exhibits phenotypic stratification (237, 238); metabolic signatures may differ significantly across these phenotypes, which is critical for the precise application of metabolite-targeted drugs.

Third, the interaction between the skin microbiome and metabolism: Staphylococcus aureus overgrows in AD lesions (239, 240), and its metabolites can directly influence epidermal metabolism and immunity; the skin microbiome in psoriasis also exhibits an imbalance (241, 242). The microbiome-metabolism-immunity triangle represents an important future research direction.

Fourth, treatment integration: Combining metabolism-targeted drugs (metformin, statins, PPAR agonists, AhR modulators, JAK inhibitors) with traditional biologics (anti-IL-17, anti-IL-13) to achieve more profound disease control in certain refractory patients is a clinical question worthy of validation (243–245).

Fifth, new metabolic targets: Novel metabolic pathways such as histone lactylation, serine-glycine metabolism, glutamine metabolism, and ferroptosis have garnered attention in inflammatory diseases (96, 246–249), but their roles in dermatology remain to be systematically explored.

Sixth, multi-omics integration and computational/AI approaches: psoriasis and AD are now among the most intensively profiled inflammatory skin diseases, and dedicated multi-omics comparisons of the two—integrating genomics, epigenomics, transcriptomics, proteomics, and metabolomics—already exist (72). The value of the immunometabolic framework proposed here is that it offers a biological scaffold on which such multi-omics layers can be aligned: the shared-node/divergent-pathway model predicts, for each node, the direction in which a given omics readout should move in each disease, turning the comparison into a set of testable, quantitative hypotheses rather than parallel descriptive catalogues. Machine-learning and other computational methods are increasingly used in this setting—for lesion classification and severity scoring, for molecular subtyping and endotyping, and for biomarker and drug-target discovery from high-dimensional omics data (250)—and are well suited to detecting metabolic patterns that distinguish the two diseases but are not apparent from any single assay. We see three concrete near-term applications: (i) supervised models that separate psoriasis from AD on the basis of lesional metabolic signatures and that flag the mixed or overlapping cases highlighted in Section 1; (ii) unsupervised clustering of metabolic-omics data to define metabolically distinct endotypes that may predict response to metabolism-directed drugs; and (iii) integrative models that link the systemic metabolic axis (Section 8), including the gut microbiome, to lesional metabolic state. Realising this will require harmonised, openly shared multi-omics datasets and careful external validation across skin phototypes and populations, without which model performance may not generalise.

## Supplementary material

Supplementary Table 1 Scoring rubric and per-item source attribution for **Figure 3**.
