# Supplementary material for: Divergent immunometabolic reprogramming in psoriasis and atopic dermatitis: a tale of two inflammatory skin diseases
Source: Front Immunol. 2026 Jul 16;17:1879634. doi: 10.3389/fimmu.2026.1879634 (PMC13422519; doi:10.3389/fimmu.2026.1879634)
Supplement: Supplementary Table 1 — Scoring rubric and per-item source attribution for Figure 3. [file Table1.docx]

**Supplementary Table S1. Scoring rubric and per-item source attribution for Figure 3.**

| **Metabolic axis / regulator** | **Th17 (PSO)** | **Th2 (AD)** | **Treg** | **Th1** | **Key reference(s)** |
| --- | --- | --- | --- | --- | --- |
| ***Panel A — metabolic preference of CD4⁺ T-cell subsets (radar)*** | | | | | |
| Glycolysis | 9 | 5 | 2 | 8 | 45, 110, 112 |
| mTORC1 activity | 9 | 5 | 2 | 7 | 45, 112, 122 |
| Amino-acid / Trp uptake (LAT1) | 7 | 4 | 3 | 6 | 168, 245 |
| Glutamine metabolism | 8 | 4 | 3 | 7 | 246 |
| Fatty-acid synthesis (ACC1/SREBP) | 7 | 6 | 2 | 4 | 113, 114, 121 |
| Fatty-acid oxidation (FAO) | 2 | 6 | 9 | 3 | 133, 134 |
| OXPHOS | 3 | 7 | 8 | 5 | 124, 125, 133, 134 |
| ***Panel B — key metabolic regulators & master transcription factors (bars)*** | | | | | |
| HIF-1α | 9 | 3 | 2 | n/a | 110, 111 |
| mTORC1 | 9 | 6 | 3 | n/a | 45, 112 |
| mTORC2 | 5 | 8 | 5 | n/a | 122, 123 |
| AMPK | 2 | 4 | 8 | n/a | 135, 136 |
| PPARγ | 3 | 8 | 7 | n/a | 120 |
| RORγt | 10 | 1 | 1 | n/a | 114, 115 |
| GATA3 | 1 | 10 | 1 | n/a | 120, 121 |
| FoxP3 | 1 | 1 | 10 | n/a | 133, 134 |
| ACC1 | 8 | 6 | 2 | n/a | 113, 121 |
| CPT1a | 2 | 5 | 9 | n/a | 133, 134 |

*Each value is an author-assigned ordinal score (0–10) graded from the cited reference(s) on the five-anchor scale defined in the Figure 3 legend (0 = absent / strongly suppressed; 2–3 = low; 5 = intermediate; 7–8 = high; 10 = dominant / defining). The scores are ranks rather than measured quantities, no primary numerical dataset underlies them, and no statistical test was applied. “n/a” indicates a subset not displayed in that panel. The table reproduces exactly the values plotted in Figure 3 (Panel A, radar; Panel B, bars).*
